# Distribution but not amount of protein intake is associated with frailty: a cross-sectional investigation in the region of Nürnberg

**DOI:** 10.1186/1475-2891-12-109

**Published:** 2013-08-05

**Authors:** Julia Bollwein, Rebecca Diekmann, Matthias J Kaiser, Jürgen M Bauer, Wolfgang Uter, Cornel C Sieber, Dorothee Volkert

**Affiliations:** 1 Institute for Biomedicine of Aging (IBA), Friedrich-Alexander Universität Erlangen-Nürnberg, Kobergerstr. 60 90408 Nürnberg, Germany; 2 Geriatric Center Oldenburg, Rahel-Straus-Straße 10, 26133 Oldenburg, Germany; 3 Institute for Medical Informatics, Biometrics and Epidemiology (IMBE), Friedrich-Alexander Universität Erlangen-Nürnberg, Waldstraße 6, 91054 Erlangen, Germany; 4 Korbinianplatz 4d, 80807 Munich, Germany

**Keywords:** Frailty, Community living older adults, Protein intake, Protein distribution

## Abstract

**Background:**

To preserve muscle mass and therefore limit the risk of disability in older adults protein intake is seen as important factor. Besides the amount of protein, its distribution over the day is thought to affect protein anabolism. This cross-sectional study investigates the association between the amount and distribution of protein intake and frailty in older adults.

**Methods:**

In 194 community-dwelling seniors (≥75 years) amount of protein intake and its distribution over the day (morning, noon, evening) were assessed using a food frequency questionnaire. Unevenness of protein distribution was calculated as coefficient of variation (CV). Frailty was defined as the presence of at least three, pre-frailty as the presence of one or two of the following criteria: weight loss, exhaustion, low physical activity, low handgrip strength and slow walking speed.

**Results:**

15.4% of the participants were frail, 40.5% were pre-frail. Median (min.-max.) daily protein intake was 77.5 (38.5–131.5) g, 1.07 (0.58–2.27) g/kg body weight (BW) and 15.9 (11.2–21.8) % of energy intake without significant differences between the frailty groups. The risk of frailty did not differ significantly between participants in the higher compared to the lowest quartile of protein intake. Frail participants consumed significantly less protein in the morning (11.9 vs. 14.9 vs. 17.4%, p = 0,007), but more at noon (61.4 vs. 60.8 vs. 55.3%, p = 0.024) than pre-frail and non-frail. The median (min.-max.) CV of protein distribution was highest in frail (0.76 (0.18–1.33)) compared to pre-frail (0.74 (0.07–1.29)) and non-frail (0.68 (0.15–1.24)) subjects (p = 0.024).

**Conclusions:**

In this sample of healthy older persons, amount of protein intake was not associated with frailty, but distribution of protein intake was significantly different between frail, pre-frail and non-frail participants. More clinical studies are needed to further clarify the relation between protein intake and frailty.

## Background

Frailty is a highly relevant geriatric syndrome which is mainly characterized by a loss of physical performance, owing to the age associated decrease of muscle mass and muscle function (sarcopenia) [1]. To preserve muscle mass and therefore limit the risk of disability in older adults, an adequate protein intake is seen as one important factor [2].

The amount of dietary protein directly affects nitrogen-balance and protein turnover. Essential amino acids are especially crucial for muscle protein synthesis. In a large cohort study in older men and women (Health, Aging and Body Composition study) over three years, lean mass decreased 40% less in the highest quintile of protein intake than in the lowest [3]. Comparing an intervention with a protein intake of 0.45 g/kg BW vs. 0.92 g/kg BW Castaneda et al. [4] found a decrease of lean tissue, muscle function and immune response in the “low protein” group after 9 weeks, whereas lean mass, muscle and immune function were preserved at the higher intake level.

Recently, an association between protein intake and physical performance has been identified in the Hertfordshire Cohort study. In this cross-sectional analysis association between higher percentage of energy from protein and faster 3 m walk was found in community-dwelling women [5]. In the InCHIANTI study, a cross-sectional study in more than 800 older Italians, the risk of being frail was twice as high in the lowest quintile of protein intake compared to higher intakes [6]. These results were confirmed by Beasley et al. [7] who described a lower risk of frailty after three years in older women with high protein intakes at baseline (Women’s Health Initiative Observational Study).

There is evidence that nitrogen turnover is not only influenced by the amount, but also by the pattern of protein feeding. El-Khoury et al. [8] found lower nitrogen excretion with a feeding pattern of three main meals compared to multiple small meals in an intervention study in healthy young adults. In older adults, Arnal et al. [9] described that a pulse protein feeding pattern (~80% of daily protein for flunch) is more efficient in improving nitrogen retention than a spread feeding pattern (four smaller meals with similar amounts of protein). The association between the distribution of protein intake over the day and physical performance or frailty has not been investigated before.

The aim of this study was to investigate the association between the amount and distribution of dietary protein and frailty in older adults.

## Methods

For this cross-sectional study 206 volunteers living independently at home were recruited from August 2009 to September 2010 in the region of Nürnberg (Germany). Potential participants were sought through a newspaper advertisement and via personal contact in a day clinic and a rehabilitation center. In order to be included, participants needed to be 75 years or older, not suffering from any illness that profoundly impacted their diet and should not show signs of significant cognitive impairment (Mini Mental State Examination ≥ 24 out of 30 points [10]). The assessments took place either at the study site or participants were visited at home, if they were not able or willing to attend the study clinic. This study was conducted according to the guidelines laid down in the Declaration of Helsinki and all procedures were approved by the ethics committee of the Friedrich-Alexander-Universität Erlangen-Nürnberg. Written informed consent was obtained from all subjects.

### Sample characteristics

The living situation was assessed as self-reported “living alone” or “not living alone”. The educational level of participants with only elementary school or no degree was defined as “low”, “medium” for those who attended a secondary school and “high” for participants with a university entrance diploma or higher degrees.

Participants’ height and weight were measured standing upright without shoes in light clothing. BMI was calculated for each subject as weight [kg]/height^2^ [m^2^].

The questionnaire on instrumental activities of daily living (IADL, 8 questions, max. 8 points) of Lawton and Browdy [11] was used to assess the degree of dependency in everyday life. A lower score designates a higher level of dependency. The participants’ answers on the IADL items dealing with dependency in going shopping and cooking meals were separately evaluated and documented as “goes shopping independently” and “cooks independently” vs. “needs help with shopping” and “needs help with cooking”. The use of medication was recorded as “more than three medications” or “less than three medications”. The Charlson Comorbidity Index (CCI) was used to assess comorbid conditions. From 19 diseases, weighted with 1, 2, 3 or 6 points, that have been found to increase mortality, a sum-score is calculated, with a higher score pointing to a higher mortality risk [12]. Reported chewing and swallowing difficulties were also documented.

### Assessment of frailty

We used the frailty definition of Fried et al. [13] and therefore assessed the following five criteria: weight loss (self-reported, more than 4.5 kg in the last year), exhaustion (self-reported feeling that everything was an effort or that one could not “get going” more than 2 times a week), low grip strength (Jamar dynamometer, men ≤ 29–32 kg, women ≤ 17–21 kg stratified by BMI quartiles of the original study sample of Fried et al. [13]), low walking speed (depending on gender and height > 6–7 sec/ 4.57 m,) and low physical activity (men < 1.6 kJ (383 kcal)/ week, women < 1.1 kJ (270 kcal)/ week) estimated with the short form of the Minnesota Leisure Time Activities Questionnaire [14]. The cut off values for grip strength, walking speed and physical activity were derived from the lowest sex specific quintiles of the original study population of Fried et al. [13]. Subjects without any of these five attributes were categorized as non-frail, those with one or two positive criteria as pre-frail and those with three or more as frail.

### Nutritional assessment

In a personal interview usual food intake was estimated using a slightly modified form of the food frequency questionnaire (FFQ) of the German part of the *European Prospective Investigation into Cancer and Nutrition*[15], which consists of 103 food items. Within this tool questions on the usual consumption of foods and food groups during the last 12 months are asked based on standard portion sizes (e. g. 1 cup, 1 piece, 1 teaspoon per month/ week/ day). Additionally there are questions on the kinds of fats used and the use of dietary supplements. The modifications mentioned above affected the definition of 12 food items to comply within our research objectives (e.g. subdividing the category “fish” into three categories of fish with different contents of fat and protein). Furthermore, the categories “bacon” and “salty snacks” were added, as they may contribute considerably to energy intake. Also a question on the consumption of unrefined cereals was added.

46 items of the FFQ were identified as main protein sources i. e. all foods derived from animal products (meat, egg, milk, fish), cereals (e. g. bread, rice, pasta) and protein rich vegetables (potatoes, legumes, soy). For these main protein sources the usual time(s) of consumption (morning, noon, evening) was asked in addition to the frequency of consumption.

From standard portions and frequencies of consumption, all items were converted to g/d. Daily energy and protein intake were calculated using the German nutrient database “Bundeslebensmittelschlüssel” (BLS II.3 [16]). Average daily protein intake is expressed as grams per day (g/d), grams per kg body weight (g/kg BW) and as percentage of daily energy intake (E%). Energy intake is expressed as kJ/d and kJ/kg BW. The amount of protein ingested per meal was ascertained by summing up the amounts of protein of the main protein sources for each meal. If more than one mealtime was indicated, an equal distribution of the portions over the indicated mealtimes was postulated.

### Data analysis and statistics

For all statistical analyses SPSS 20.0 (IBM) software was used.

Sample characteristics are presented as median (min-max.) for continuous variables and as percent for categorial variables. The distribution of the prevalence of participants’ characteristics in the three frailty groups was tested for significant differences by χ^2^ testing.

Differences in continuous sample characteristics, daily protein intake (g, g/kg BW, E%) as well as in distribution of protein intake (%) over the three mealtimes in non- frail, pre-frail and frail participants were tested for significance by Kruskall-Wallis test. A coefficient of variation (CV = SD/ mean value) of protein intake (g/meal) in the morning, at noon and in the evening was calculated for every participant to estimate the unevenness of the distribution of protein intake over the day. The CV is a dimensionless, relative measure of statistical dispersion. A CV of zero connotes a total evenness of the protein intake over the day, i. e. the same amount of protein is ingested in the morning, at noon and in the evening. The more uneven the distribution is, the higher is the individual CV of protein intake. CV is presented as median (min.-max.) for each of the three frailty groups and compared by Kruskal-Wallis testing. Distribution of the CV of protein intake in the single, dichotomous frailty criteria was compared by Mann-Whitney-U testing.

The risk of being frail or pre-frail vs. non frail and the risk of each single frailty criterion, respectively, in the 2nd, 3rd and 4th quartile of protein intake (g/kg BW) vs. the 1st quartile (lowest intake) was calculated as odds ratios (OR) accompanied by 95% confidence intervals by multinomial logistic regression analyses. Confounding covariates were identified by ‘manual backward elimination’ with exclusion if an initially included factor was both insignificant and did not cause a change-in-estimate of > 10% of the exposure of interest.

## Results

194 study subjects (68 men and 127 women) providing complete information (less than three items missing) on the FFQ were included in the following analysis. Participants had a median age of 83 (75–96) years. Pre-frailty was found in 40.5% of the participants and 15.4% were frail.

The three frailty groups differed significantly in the distribution of sex (P < 0.05) and age (P < 0.001) (Table 1). Frail participants lived alone more often than pre-frail and non-frail (P < 0.05) and had a lower educational level. Median BMI was 27.1 (18.6-36.1) kg/m^2^ without significant differences between the three groups. Frail participants scored significantly higher on the CCI and were more likely to use more than three medications than pre-frail and non-frail persons (P < 0.05). Chewing and swallowing difficulties were significantly more prevalent with increasing frailty status. Median daily energy intake was 8.5 (4.4–14.9) kJ without differences between the three frailty groups (Table 1).

**Table 1:** Main characteristics of the population (n = 194)

	**Non-frail (n = 85)**	**Pre-frail (n = 79)**	**Frail (n = 30)**	**Total**	**P**
Female sex^†^ (n = 128)	57.0	65.8	86.7	66.0	0.013^a^
Age [years] ^‡^	82 (76–91)	84 (76–94)	86 (75–96)	83 (75–96)	0.000^b^
Living alone (n = 122)	50.6	59.5	80.0	63.3	0.025
Educational level^§^					0.005
low (n = 83)	42.3	39.2	53.3	42.8	
medium (n = 59)	23.5	31.6	46.7	30.4	
high (n = 52)	34.1	29.1	0.0	26.8	
BMI [kg/m^2^]	26.7 (21.0–35.0)	28.1 (20.9–35.3)	26.2 (18.6–36.1)	27.1 (18.6–36.1)	0.11
MMSE [points]	29 (25–30)	29 (24–30)	29 (25–30)	29 (24–30)	0.42
IADL [points]	8.0 (5.0–8.0)	8.0 (1.0–8.0)	7.0 (2.0–8.0)	8.0 (1.0–8.0)	0.000
Goes shopping independently (n = 151)	96.5	87.3	50.0	77.9	0.000
Cooks independently (n = 158)	88.4	82.3	73.3	81.3	0.13
More than 3 medications (n = 101)	35.7	51.9	69.0	52.2	0.005
CCI [points]	0.0 (0.0–5.0)	1.0 (0.0–3.0)	2.0 (0.0–4.0)	1.0 (0.0–5.0)	0.001
Chewing difficulties (n = 74)	10.7	26.6	40.0	38.0	0.001
Swallowing difficulties (n = 16)	2.4	12.7	10.0	8.3	0.044
Energy intake [kJ/d]	8.8 (4.4–12.6)	8.5 (4.6–14.9)	7.9 (3.4–13.7)	8.5 (4.4–14.9)	0.32
Energy intake [kJ/kg BW]	0.12 (0.07–0.22)	0.12 (0.05–0.23)	0.13 (0.07–0.20)	0.12 (0.05–0.23)	0.60

*BW* body weight, *CCI* Charlson Comorbidity Index [12], *IADL* Instrumental Activities of Daily Living [11], *MMSE* Mini Mental State Examination [10].

^a^ Chi-square Test (for all categorial variables) ^b^ Kruskall-Wallis Test (all continuous variables).

^†^ In percent (all categorial variables) ^‡^ Median (min.-max.) (all continuous variables) ^§^ “low” = elementary school or no degree; “medium” = secondary school; “high” = university entrance diploma or higher degrees.

Median (min.-max.) daily protein intake was 77.5 (38.5–131.5) g, 1.07 (0.58–2.27) g/kg BW and 15.9 (11.2–21.8) E%. A trend in the amount of protein ingested could not be identified with increasing frailty status (Table 2). Accordingly, we found no differences in the risk of frailty or its single criteria in quartiles of higher protein intake compared to the quartile with the lowest intake. We only found a significant p for trend concerning low physical activity (Table 3).

**Table 2:** **Amount**^**a **^**of daily protein intake in three frailty groups as g/day, g/kg BW and E%**

**Frailty**	**Non-frail (n = 86)**	**Pre-frail (n = 79)**	**Frail (n = 30)**	**P**^**b**^
g/day	77.4 (39.0–113.4)	78.3 (38.5–131.5)	74.1 (44.3–117.9)	0.12
g/kg BW	1.06 (0.63–1.75)	1.09 (0.58–2.27)	1.07 (0.58–2.00)	0.68
E%	15.5 (12.0–21.4)	16.4 (12.0–21.8)	15.1 (11.6–18.5)	0.039

*BW* body weight, *E%*, percent of energy intake.

^a^ Data are given as [median (min.-max.)], n = 194.

^b^ Kruskall-Wallis test.

**Table 3:** **Risk**^**a **^**of frailty, pre-frailty and of the single frailty criteria in the quartiles of protein intake [g/kg BW]**^**b**^

	**n**	**1 (<=0.90)**	**2 (0.91–1.07)**	**3 (1.08–1.27)**	**4 (>1.27)**	**P trend**
**Frailty**	30	1.00	4.27 (0.66–27.60)	1.27 (0.22–7.74)	1.90 (0.36–9.88)	0.887
**Pre-frailty**	79	1.00	0.81 (0.30–2.24)	0.60 (0.23–1.56)	1.64 (0.50–5.35)	0.648
Weight loss	16	1.00	0.93 (0.18–4.78)	1.03 (0.20–5.31)	0.43 (0.09–2.18)	0.394
Exhaustion	43	1.00	2.02 (0.66–6.18)	1.10 (0.34–3.48)	1.76 (0.63–4.94)	0.428
Low hand grip strength	76	1.00	0.86 (0.33–2.28)	0.34 (0.13–0.88)*	0.70 (0.27–1.79)	0.182
Slow walking speed	43	1.00	5.66 (1.17–27.42)*	2.13 (0.48–9.58)	4.19 (0.94–18.71)	0.188
Low physical activity	39	1.00	0.90 (0.30–2.66)	0.43 (0.14–1.36)	0.37 (0.12–1.13)	0.021*

^a^ Odds ratio (95% confidence interval), calculated by multinomial logistic regression.

adjusted for age and sex, IADL score, number of medications, and chewing difficulties.

^b^ with first quartile as reference indicating individuals with lowest protein intake.

*p < 0.05.

The main protein sources covered a median (min.-max.) of 73.8 (45.7–90.5) % of total protein intake. Most of this protein was ingested at noon (60.2 (0.0–84.5)%), about one fourth (25.1 (0.2–70.5)%) in the evening and 15.3 (0.0–47.4) % in the morning. With increasing frailty, the percentage of protein ingested in the morning decreased significantly, whereas it increased at noon (Table 4).

**Table 4:** Percentage of protein ingested in the morning, at noon and in the evening in three frailty groups [median (min.-max.)]

**Frailty**	**Non-frail (n = 86)**	**Pre-frail (n = 79)**	**Frail (n = 30)**	**P **^**a**^
% morning	17.4 (2.8–47.4)	14.9 (0.0–43.1)	11.9 (0.0–29.8)	0.012
% noon	55.3 (16.9–79.9)	60.8 (0.0–83.0)	61.4 (31.6–84.5)	0.041
% evening	24.3 (0.2–39.2)	25.4 (0.4–70.5)	23.6 (7.3–55.4)	0.944

^a^ Kruskall-Wallis test.

**Figure 1:**
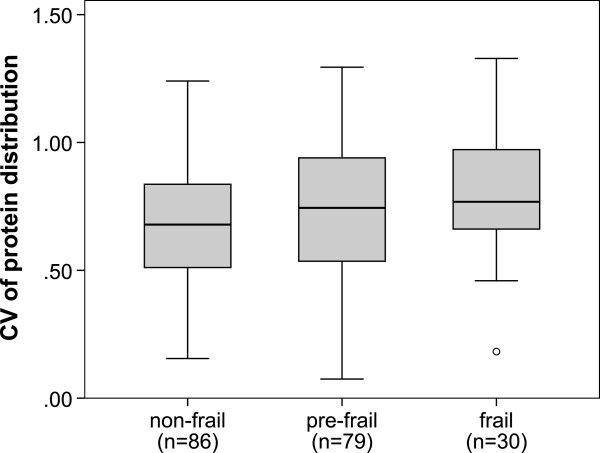
**Boxplots of coefficient of variation (CV) of protein distribution over the three daily mealtimes in non-frail, pre-frail and frail participants.** The boxes represent the interquartile range with the bold horizontal lines denoting median CV. The whiskers show the highest and lowest values within 1.5 box lengths from either end of the box and the circles represent outliers. Kruskall-Wallis testing showed that the median CV differed significantly between the three frailty groups (P < 0.05).

The median CV of frail (0.77 (0.18–1.33)), pre-frail (0.74 (0.07–1.23)) and non-frail (0.68 (0.15–1.24)) persons differed significantly (P < 0.05) (Figure 1). The CV was significantly higher in subjects with low walking speed and exhaustion (P < 0.05) than in participants without these impairments (Table 5).

**Table 5:** Coefficient of variation (CV) of protein distribution over the three daily mealtimes [median (min.-max.)] in participants with and without the single frailty criteria

**Frailty criterion**	**n**	**Criterion is present**	**Criterion is not present**	**a**
Weight loss	16	0.78 (0.46–1.21)	0.73 (0.07–1.33)	0.263
Exhaustion	43	0.83 (0.18–1.33)	0.72 (0.07–1.29)	0.041
Low hand grip strength	76	0.76 (0.07–1.33)	0.72 (0.15–1.24)	0.109
Slow walking speed	43	0.87 (0.18–1.26)	0.70 (0.07–1.33)	0.002
Low physical activity	39	0.74 (0.07–1.33)	0.73 (0.15–1.29)	0.732

a Mann-Whitney-U test.

## Discussion

In this study no differences were found in the amount of protein intake between frail, pre-frail and non-frail community-dwelling older adults. With regard to the distribution of protein intake, frail subjects showed a different and more uneven distribution of their protein intake over the day with lower intake at breakfast and higher intake at lunch. To our knowledge this is the first study investigating the association between the distribution of protein intake and frailty.

Contrary to Bartali et al. [6], who found an association between low protein intake (lowest quintile) and frailty in a cross-sectional analysis of the InCHIANTI study, in our sample the risk of frailty was not reduced in the quartiles of higher intakes compared to the quartile of the lowest protein intake. Furthermore, there is some evidence on the association of protein intake with walking speed [5] as well as handgrip strength [17], which we could also not identify in our analyses. This might be due to the relatively high protein intake even in the lowest quartile, where the cut off was ≥0.9 g/kg BW protein/day. This is above the value of 0.8 g/kg which is the present recommendation for protein intake in younger as well as in older adults [18], and has been found to be a threshold for negative nitrogen balance [19], low muscle mass [20] and more health problems after 10 years [21].

Our study is the first to investigate the association between the distribution of protein over all the meals and frailty. Regarding evenness of this distribution we found a more even protein eating pattern in non-frail participants than in frail and pre-frail (Figure 1) and in those reporting exhaustion and slow walking speed (Table 5). These results are in line with the recommendations of Paddon-Jones et al. [22] to equally distribute the daily protein intake of older adults over breakfast, lunch and dinner. Arnal et al. [9], in contrast, found one large serving of protein (80% of daily protein intake in one of three meals) to be more effective to promote a positive nitrogen balance and muscle synthesis in older adults than protein intake evenly spread over four meals. This discrepancy may be explained by the low absolute amount of daily protein administered to Arnal et al.’s participants (approx. 65 g). That means that in the spread feeding group a single meal contained hardly 20 g of protein, the minimum needed for stimulating muscle protein synthesis according to Paddon-Jones et al. [22], whereas with a higher total daily protein intake, as found in our sample, an even distribution results in more meals containing at least 20 g protein. At the same time, fasting losses between meals, which increase with the amount of protein of the meal [23], are reduced in a more even distribution. Finally, comparing our data with trials on nitrogen balance and muscle protein synthesis we must be aware of the fact that these results may not be totally transferable to issues of physical performance and frailty.

For the assessment of usual dietary habits, we used the FFQ of the German part of the EPIC study, which is well validated [24]. A limitation of this questionnaire is the proven underreporting of energy and protein intake of about 20% [24]. Thus, actual protein intake in our participants is supposedly higher. The validity of the results on distribution of protein intake are limited by the fact that although our 46 *a priori* set of main protein sources obviously covered all important sources of actual dietary protein, they still left an average of 26.2% of dietary protein undocumented. This may be due to some of our participants consuming very small amounts of protein, mainly from vegetable foods that were not considered as main protein sources, e. g. fruit (especially bananas) and vegetables (especially tomato sauce).

We are aware of the limitation that we only assessed protein intake at the main meals and only for selected foods. This was decided to avoid an undue length of the FFQ, which would probably have overstressed our aged participants. On the other hand Roussett et al. [25] found in a cross-sectional study in healthy older adults, that snacks only contributed to 1.4% in men and 2.3% in women to protein intake. Therefore snacks were seen as negligible in our distribution analysis. Up to now, there is no common method for evaluating protein distribution over all meals. Using the CV as measure for unevenness of distribution can be seen as a first approach. Certainly, assessment tools have to be developed to enable a more detailed evaluation of meal habits and statistical methods have to be adapted to further clarify the impact of protein distribution on sarcopenia, physical performance and frailty.

A major limitation of the study is its cross-sectional design which does not allow any statements on causal relationship. It is also plausible that frailty vice versa affects protein intake, for example by impairments in going shopping, chewing or swallowing (Table 1). Another critical point is that we did not consider the influence of protein quality on frailty, although it is an important regulator of protein metabolism. The small sample size, especially in the group of frail participants limits the study’s statistical power. Nevertheless, we detected significant associations. Furthermore, the sample consists of volunteers and may therefore lack in representation of the older German population in general.

A strong advantage of our study is that all FFQ have been conducted in personal interviews by a single experienced nutritionist, and for all assessments well validated tools have been used. This is the first study investigating the distribution of protein intake in German older adults and the association between this distribution and frailty. Unique is the development of a parameter of unevenness of protein distribution, which to our knowledge has not been used before in this setting.

In summary, in our study group of very old independently living senior citizens only few were frail and even the lowest quartile of protein intake was above the recommendation of 0.8 g/kg BW. There was no significant difference in the amount of protein ingested between frailty groups and we found no reduced risk for frailty in the quartiles with a higher protein intake compared to the lowest quartile. Our results also showed a relation between frailty and the distribution of daily protein intake over the main meals. Studies on the effect of protein intake on functional and clinical outcomes are still scarce. Therefore we recommend further investigation on this topic on the basis of the results of our study.

## Abbreviations

BLS: Bundeslebensmittelschlüssel (national food database); BW: Body weight; CCI: Charlson comorbidity index; CI: Confidence interval; CV: Coefficient of variation; EPIC: European Prospective Investigation into Cancer; E%: Percent of energy intake; FFQ: Food frequency questionnaire; IADL: Instrumental activities of daily living; MMSE: Mini mental state examination; OR: Odds ratio.

## Competing interest

The authors declare that they have no competing interests.

## Authors’ contributions

The authors’ responsibilities were as follows: DV, JMB and CCS designed the research. JB, MJK and RD conducted the research. JB analyzed the data and performed statistical analyses. WU supervised the statistical analysis. JB drafted the manuscript with appreciable input from DV. JB and DV had prime responsibility for the final manuscript content. JMB, MJK, CCS, WU also contributed to the final manuscript. All authors read and approved the final manuscript.
